# Odontological Society

**Published:** 1888-05

**Authors:** 


ARTICLE V.

### ODONTOLOGICAL SOCIETY.

The ordinary monthly meeting of the above Society was held on the 6th ult. at 40, Leicester Square, Mr. S. J. Hutchinson, (Vice-President) in the chair.

Mr. Hutchinson read a letter from the President, Mr. Daniel Corbett, regretting that ill health and bad weather prevented his attendance, and hoping that he wou'd be able to be present to read his Inaugural Address at the March meeting.

The Librarian reported several presentations to the library, including Mr. Christopher Heath's Lectures on "Certain Diseases of the Jaws;" "Note Book for Dental Students," by Mr. James Rymer; "The Proceedings of the Royal Institution;" "Footprints of the Profession," being an Address to the Maine Dental Society; and two pamphlets by Dr. Magitot.

The Curator said that they had added a very fine specimen of a Tetradont to their collection. Mr, Howard Mummery presented the skull of a rabbit showing injury to the pulps of incisor teeth some long time previous to death. There was also a case of dilaceration of the incisor teeth of a porcupine, which was similar to the rabbit just mentioned.

Dr. W. St. George Elliott made a "casual communication" on "A System of Crowns." Previously he had had something to say on the advisability of crowning teeth. Many, doubtless, had looked into the subject, and those who had done so would certainly have recognised that it was advisable. He was of opinion that of all operations there was none which gave so much satisfaction both to patient and operator, a statement which could hardly be made of bridge-work and some other methods. It would be remembered that Dr. Knapp wrote an article for the *Dental Cosmos*, in which he described an elaborate system for making crowns. When Dr. Knapp was in London he gave a demonstration, at Dr. Elliott's invitation, and it was considered very successful. But while the exceedingly artistic character of the work was admitted, the length of time required (five hours) made it a somewhat impracticable operation, the crown being entirely of gold. Dr. Elliott had adopted a method which was a great saving of time, and he claimed for it equal advantages in point of efficiency with Dr. Knapp's system. It was somewhat allied to the principle of the Richmond crown, the cardinal feature of which was the fixing a gold cap or lid upon the stump of the tooth, the stump having been cut away nearly to the alveolar margin of the gum. But there was the objection to this plan that the gold was frequently visible. Dr. Elliott recommended his own process as exceedingly simple. In the first place it was not necessary, as by the Richmond method, to cut away the whole of the remaining portion of the stump. He always allowed every portion of the tooth remaining to assist in holding the crown, providing it was not in the way. An impression of the stump and the bite was taken, then a process similar but not exactly the same as suaging was followed, which gave a cap the exact copy of nature.

Mr. Redman (of Brighton) could fully endorse, from his own practical experience, all that Dr. Elliott had said, but found the best plan for getting a proper occlusion plate was by Dr. Knapp's process.

Dr. G. Cunningham (Cambridge) said, with regard to the system of capping the crown, he confessed that while æsthetically the system recommended by Dr. Elliott looked better, for getting thorough occlusion nothing could excel, in his opinion, the use of the gold ferrule without the cap, and with or without How's screws in the root canals, and filling up the cavity with amalgam, With reference to taking a model of the root, he had lately adopted a modification of the Herbst method for using the matrix, and thought it better than the wire method.

In connection with the system of using ferrules in crowning teeth, there was one point in its favour—viz., that the ferrule made the operation of crowning possible in many cases which had passed beyond the stage where the pivot method was applicable.

Dr. Mitchell regarded anything in the way of models as a waste of time, and described his own method of striking up tops, by which he was able to reduce the time taken to produce a gold crown from five to one or two hours. He thought that two hours was long enough for any operation in gold crowning.

Mr. J. Smith-Turner was very much struck with some of the views expressed on the subject. It seemed to him that the operation of cutting away part of the tooth, putting a ferrule round the root and pushing it well down to the gum, instituted that very condition of things which they were told to avoid when plugging a tooth.

Dr. Field: Perhaps Mr. Smith-Turner forgot that in filling or plugging a tooth they were in most cases dealing with teeth not very far advanced in decay, whereas in crowning the tooth they were dealing with the merest shell.

Dr. Mitchell remarked that in the case of the stopping or filling they had more or less rough material upon the gum; in the other they had a highly polished bank.

Dr. Cunningham said that, like Mr. Smith-Turner, he had been brought up in the orthodox school, but having seen very successful results from Dr. Elliott's method, and practically experienced the advantage of it, his first prejudices had been overcome. He thought the answer to Mr. Smith-Turner was that the periosteum did not descend down to the neck of the tooth for some considerable way.

Mr. Smith-Turner was still of opinion that his position had not been assailed.

Dr. Elliott said that, in the first place, his was not a new-fangled notion. He had seen crowned teeth fifteen years ago. He had never met with failure of a crown nor any dental irritation.

With regard to the nitrous oxide blow-pipe, Dr. Rollo Knapp claimed to be the inventor, and he patented the invention both in America and in England. Whether his conduct had been in accordance with their notions of propriety, he (Dr. Elliott) would not say. But this he would say, that one of the members of the Odontological Society, Mr. Hunt, of Yeovil, used and described the use of the nitrous oxide blow-pipe nineteen years ago for melting gold. He would add, however, that the whole thing was very simple, a bottle of nitrous oxide and an ordinary blow-pipe being all that was required.

Mr. Bland Sutton read a short paper in connection with his previous paper on "The Classification of Odontomes." Two days after reading that paper a "thar," or Himalayan goat, died in the Zoological Gardens with a large suppurating mass involving each upper jaw. Beneath the right orbit existed a sinus discharging pus. The upper molar teeth were defective, and in the middle of each alveolar border there was a large recess leading into the antrum. These recesses contained a considerable quantity of fine chaff and hay. A longitudinal bisection showed that each antrum contained a cyst with dense thick walls, the outer shell being of bone, lined with thick fibrous tissue, and the interior being filled with denticles, fragments of cementum, and bone of varying sizes amounting to nearly 300.

A careful study of the specimen had convinced him that all those remarkable cases included in his list as "anomalous" odontomes had their origin in aberrant follicles of that nature. He therefore proposed to include "anomalous odontomes" under the term "Compound Follicular Odontomes" in section B of his previous paper. The case further justified him in including dentigerous cysts among odontomes. Having described the characteristics of Follicular Cysts, Fibrous Odontomes, and Cementomas, he concluded by saying that the specimen which formed the subject of the paper was further interesting as confirming his opinion that the hard tumours seen in museum, only represented half the case. Every odontome had a periodontomal capsule on which it depended for nutrition. When the cysts suppurated the capsule became destroyed, the hard part necrosed, and in ordinary cases sloughed out. Odontomes were practically teeth, and like teeth, underwent eruption. The suppuration usually attendant upon this was frequently the first indication of trouble.

Mr. S. J. Hutchinson asked if many of the denticles contained true cementum, and if any contained dentine.

Mr. Bland Sutton replied that some contained cementum, not many, and it was uncertain whether any contained dentine.

Mr. C. S. Tomes said he would like to ask Mr. Bland Sutton a question with regard to this most remarkable and completely described odontome. Mr. Sutton said that in it there appeared a mass which resembled alveolus. Did not this put it in the classification of ovarian tumours, where there would be a large amount of bone formation?

Mr. Bland Sutton replying, said that in all those cases in which dentigerous ovarian cysts containing teeth had been described, skin containing hairs, muscle, &c., might be associated with them, but in the present case none of the characteristics of ovarian tumours presented themselves.

Mr. Storer Bennett begged leave to point out that there was rather a closer connection between ovarian tumours and odontomes than was supposed. Mr. Sutton had said that ovarian tumours had hair in connection with them. It so happened that he (Mr. Storer Bennett) had met with an odontome taken from a pig in which a number of bristles were distinctly visible.

Mr. Bland Sutton thought that Mr. Storer Bennett had probably mistaken whisps of chaff, grass, or hay for hairs.

Dr. A. W. Harlan, of Chicago, then read a paper on

### THE TREATMENT OF PULPLESS TEETH FROM THE POINT OF VIEW OF EVERYDAY PRACTICE.

Dental surgeons were everywhere devoting much of their thought, ingenuity and energy in the direction of maintaining within the mouth many pulpless teeth that in former times were needlessly extracted.

Out of this growing conservative tendency had grown various procedures; some designated by their authors "mechanical methods," and others "the immediate dressing and filling method," and still others as "the time dressing, or therapeutic expectant, method." This variety of treatment would be a danger to the student and inexperienced surgeon unless directed by a strong hand.

Pulpless teeth were found in all the "seven ages of man;" even the teeth of the first dentition did not escape the ravages of caries, nor was slow death of the pulp, or its destitution by other means, unfrequent.

Pulpless deciduous teeth and roots might properly be retained for mastication up to or about the time of the expected eruption of their successors.

For the convenience of handling the subject, he would divide it under the following headings:—1, teeth deprived of their pulp by means of corrosive medicaments, or immediate operative procedure, cautery, broaches, wooden points, anæsthesia, or accident; 2, death of the pulp as a result of capping from rapid thermal changes; 3, death from partial laceration, as from a blow or fall, or from torsion in correcting irregularities, or from imperfect or partial extractions and replacement of teeth; 4, death from gradual exposure by caries or some form of abrasion or erosion; 5, death from exposure through salivary deposits, exposing the apices of roots by displacement of tissues, or from the development of pockets resulting from the so-called "Riggs' disease" and their extension to or around the apices of roots; or death by irritation due to pulp nodules, or strangulation, and from unknown causes.

The classification, imperfect as it was, would suggest that no single method would suffice to meet all the various pathological conditions.

From a somewhat extended experience, covering a period of nearly twenty years, he had a record, not of hundreds, but of thousands of teeth treated and their roots filled with few recurrences of fistulæ or abscesses.

His method of procedure was, for Class 1, three things to be insisted upon. (*a*) Complete disinfection, nothing being allowed to enter the pulp chamber that was not placed there by his own hands, by means of clean and sterilized instruments. Water and saliva to be rigidly excluded. (*b*) No powerful coagulators of albumen to be introduced into the pulp chamber prior to removal of the pulp. (*c*) Any incorporation of antiseptics or disinfectants with the filling to be avoided. Having destroyed the pulp by any of the methods referred to, extirpation followed; in the case of fractured teeth cessation of hemorrhage should be waited for. Then the pulp chamber should be syringed with equal parts of peroxide of hydrogen and a 1-1000 solution of bichloride of mercury until the canals were free from blood and serum. The root should then be dessicated with absolute alcohol and heated air, and filled at once. If the pulp had been destroyed by arsenic or similar agent, a dressing of tannin and glycerine should be applied to the devitalized pulp, first puncturing it to allow any congested blood to escape. The dressing should be covered with gutta-percha, perforated in two or three places, to allow of the escape of confined air. After eight days the dressing should be removed, the rubber-dam placed over this tooth, and the pulp extirpated with suitable sterilized broaches. The pulp chamber should then be thoroughly washed with the solution mentioned, and the root filled at once. The roots should not be drilled, but access obtained by freely opening the pulp chamber.

Class 2. There was little difficulty in diagnosing this class, one of three conditions always being present, viz., encystment of the apex, a dormant or cold abscess, or a fistulous tract leading from the end of the root externally.

*Treatment.*—For encystment, disinfecting dressing and immediate root filling. For dormant abscess, the rubber-dam being adjusted, the tooth and drill should be bathed in terebene, the pulp chamber entered, and the contents at once bathed with a penetrating disinfectant, which should be allowed to remain for a few minutes. Roots should not be drilled unless they were carious. Cotton wool saturated with some essential oil should be packed lightly in the root and the pulp chamber filled with dry cotton, over which should be packed some soft gutta-percha perforated for the escape of gases. After ten days the dressing should be removed. Absence of gas or moisture would show whether the abscess had disappeared. Either being present, the treatment should be repeated. The value of an essential oil dressing was that the reproduction of tissue was stimulated.

The introduction of a powerful coagulator of albumen would be most disastrous without providing for drainage. It would be far better to cut through the gums and alveolar process and provide an outlet at once. Time would always be a factor in the destruction of microbes, and the disinfection of infected dentine was a matter of no small importance. The practice of leaving antiseptics and disinfectants within the root canal or pulp chamber as a safeguard against future trouble was delusive. In cases of fistulæ, disinfection and immediate filling was generally possible.

Class 3. Treatment same as Class 2.

Class 4. In death of the pulp from abrasion or erosion, in nearly every instance the roots would be found to be encysted; in such cases immediate filling might be proceeded with after thorough disinfection.

Class 5. In this class immediate filling was not desirable when the teeth were to be retained. Dr. Harlan advised cleansing the canals as in Canal 1, then packing the roots with threads of cotton wool saturated with a mixture of olei cinnamoni 20 minims, olei gaultherii, terebin. *aa* 30 minims, and seal the crown cavity with hard gutta-percha. The dressing should be allowed to remain from one to six months, and the root then filled permanently.

Class 6. Circumstances generally pointed to the necessity of temporary root dressings to prevent pain and swelling.

Dr. Harlan, in conclusion, said he was not unmindful that certain complications might and did arise. Certain relief was always afforded by drilling directly into the pulp chamber, a course to be preferred to drilling through the alveolar process.

After entering the pulp chamber and gently removing the pulp under antiseptic conditions, great comfort would be given to the patient by painting the dried gum with equal parts of chloroform, tincture of aconite and tincture of iodine; and the immediate administration of 1-10th gr. pills of calcium sulphide every fifteen minutes, until four or six had been taken. Occasionally pulpless teeth were elongated, and closing of the jaws gave pain. This might be obviated by grinding the occluding serfaces. Much of the success in the management of pulpless teeth Dr. Harlan attributed to the thorough removal of all pulp tissue and other foreign matters, and the fidelity of filling the roots with a substance neither porous nor corruptible by the fluids of the mouth.

The Chairman said Dr. Harlan had brought forward the subject in such a masterly manner that he felt sure many of the members would be desirous to speak upon it, and he trusted that as there was not much time at their disposal for discussion, they would not allow the momento to slip by unutilised.

Dr. Mitchell strongly supported the part of the paper which laid special stress upon the importance of cleanliness and regarded it as one of the important factors towards success. With reference to the common practice of filling teeth with antiseptic dressings, he had known cases where it had been necessary to refill such teeth, and the offensive odour omitted from the removed dressing was a sufficient condemnation of the practice There was nothing like doing a thing thoroughly that was worth doing at all.

Dr. St. George Elliott showed and described a combined syringe and washing apparatus for treating and washing dead teeth. It was made with a spring, had an ordinary nossle for ordinary use, and also two platina nossles. By means of this syringe it was possible to pass water backwards and forwards in the nerve canal until it was thoroughly cleansed. There was a still finer nossle which Dr. Elliott claimed could be passed two-thirds of the way up the canal and remove the greater part of the *debris.* He did not see why it should not be possible to pass gutta-percha up the canal and allow it to do its own filling.

Mr. C. S. Tomes thought that in their daily practice in dealing with a septic condition of the teeth they ran some danger of losing sight of what they had to do with. He was by no means sure that Dr. Harlan, in his free use of antiseptics, had not once or twice done so. In the first place, during operations in the mouth, an aseptic condition was almost impossible. He wished particularly to call attention to the point that they were told to sterilize their instruments, but if anyone hoped to puncture with an antiseptic, let him try how far an ordinary instrument would pierce into the dentine, and they would find it would make very little headway indeed. Dr. Harlan mentioned the necessity of observing a series of precaution in cleaning out the tooth; then he put the stopping in and covered it with gutta-percha, in which he bored holes. He (Mr. Tomes) thought that in treating such a tooth they would do well to dismiss from their minds the possibility of attaining perfect aseptic conditions. He wished merely to sound a note of warning, not to talk about something impossible of attaining, and forget more practical considerations, which would lead to more practical success.

Dr. Field had had some good rrsults in his own practice by the adoption of the method set forth by Dr. Harlan, but with occasional failures for which he could not account, which would discover themselves sometimes after three months, sometimes after three years. He was of opinion that success depended on delicacy of manipulation, not permitting the fine-pointed instruments to puncture beyond the apex of the root. He wished to say a word in support of the old method. He had seen the roots of teeth filled with nothing but cotton wool and creosote that had stood for years, so that in point of fact, although complete, antiseptic conditions were not obtainable. The closure of the foramen had been obtained, which led him to the conclusion with which he started—viz., that whether they filled with one material or another, the main point seemed to be delicacy of manipulation. If this were secured, he believed they might use half-a-dozen different filling materials and obtain satisfactory results.

Dr. Walker said perhaps it would afford the Society some little pleasure to be reminded that the subject dealt with by the paper was no new thing; that so far back as 1865 their then President, their present Treasurer, Mr. Thos. Arnold Rogers, who was with them that evening, and would no doubt have spoken but that he was very unwell, read a paper on that very subject, and any member who felt interested in the subject, by turning to the volume of the "Transactions" for 1865, could read for himself. Mr. Rogers there gave a long account of how he filled and treated roots; he showed his instruments to the Society and gave them records of his own treatment. He (Dr. Walker) had the pleasure of co-operating with him at that time, and it was only a few weeks ago that the speaker saw a canine he had plugged in 1871 by the method advocated in 1865 which was then in good condition. He had followed out the plan of not filling with cotton but with metal to the apex of the root, and he fully bore out Dr. Harlan's view that if sound roots were filled with metal good results would be obtained. He had seen Dr. Harlan's work in Chicago, and he was struck with the very great care he exercised, especially in preventing moisture returning to the roots after treatment.

The discussion was continued by Messrs. T. A. Rogers, Hern, Walter Harrison, Lloyd Williams and R. H. Woodhouse.

Dr. Harlan, in replying, said with reference to the remarks of Mr. Tomes, he felt sure that they would agree with him that in order to protect gelatine or any other substance from microbes and their spawn, the cotton wool was the very best substance to stop the tubes with, and it would not matter if the cotton wool should not be dry. It was not expected that the wool would remain dry for more than a moment, with the perforation in the gutta-percha cap; but it would intercept and prevent any bacterium flowing into the pulp-chamber. Mr. Hern appeared not to have followed the portion of the paper which dealt with cleansing and disinfecting. He objected to his (Dr. Harlan) using essential oils, saying the oils were incompatible with water. If Mr. Hern had followed the paper carefully, he would have seen that the parts were cleansed with peroxide of hydrogen or corrosive sublimate solution and thoroughly dried. Then again, when a strand of cotton wool was interposed, saturated with some essential oil below body heat, he would agree with him that that became at once a non-irritating and permanent disinfectant, as distinct from an antiseptic as a disinfectant could be. He was afraid he was unable to agree with Mr. Hern in reference to disinfecting by mere drilling. He would reply to Dr. Walker, that the practice of drilling or reaming roots would never become universal. With reference to wooden points, he would remind Dr. Cunningham that he (Dr. Harlan) had published eight cases, and he was bound to say that six out of the eight patients had threatened to leave him never to return.

After the usual vote of thanks, the Society adjourned.
—*London Dental Record.*

---

